# Geosynthetics in geoenvironmental engineering

**DOI:** 10.1088/1468-6996/16/3/034605

**Published:** 2015-05-08

**Authors:** Werner W Müller, Fokke Saathoff

**Affiliations:** 1 BAM Federal Institute for Materials Research and Testing, Unter den Eichen 87, D-12205 Berlin, Germany; 2 Chair of Geotechnics and Coastal Engineering, Universität Rostock, Justus-von-Liebig-Weg 6, LAGII, D-18059 Rostock, Germany

**Keywords:** geosynthetics, geoenvironmental engineering, landfill capping system, coastal protection, geocomposite drain, geogrid, geotextile

## Abstract

Geosynthetics are planar polymeric products, which are used in connection with soil, rock or other soil-like materials to fulfill various functions in geoenvironmental engineering. Geosynthetics are of ever-growing importance in the construction industry. Sealing of waste storage facilities to safely prevent the emission of wastewater, landfill gas and contaminated dust as well as the diffusion of pollutants into the environment and coastal protection against storms and floods and reconstruction after natural disaster are important fields of application. We will give an overview of the various geosynthetic products. Two examples of the material problems related to geosynthetics are discussed in detail: the effect of creep on the long-term performance of geocomposite drains and the numerical simulation of the interaction of soil with geogrids. Both issues are of importance for the use of these products in landfill capping systems. The various functions, which geosynthetics may fulfill in the protection of coastal lines, are illustrated by case studies. The geosynthetic market is evaluated and economical and environmental benefits, as well as environmental side effects related to the use of geosynthetics, are discussed.

## 1. Introduction: what are geosynthetics?

**Figure 1.**
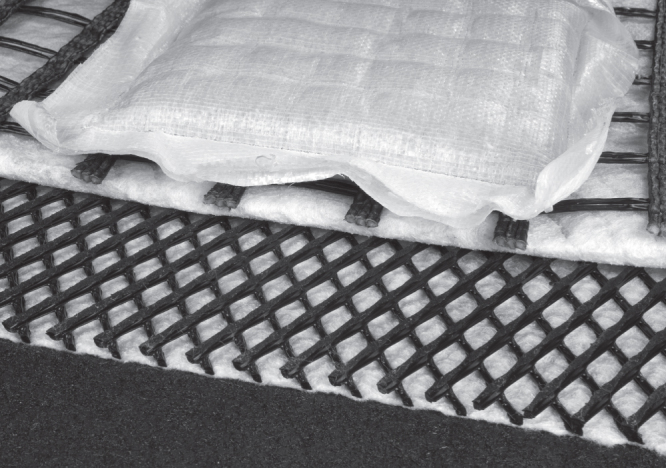
Various samples of geosynthetics. From bottom to top: rough black surface of a high density polyethylene (HDPE) geomembrane, white carrier nonwoven geotextile, black geonet, white nonwoven filter geotextile (folded back), black woven geogrid and a white sand-filled geocontainer made of a nonwoven.

Geoenvironmental engineering is the engineering discipline that deals with the application of geotechnical methods to the solution of environmental problems. Examples are remediation of contaminated sites, water resource engineering, wastewater treatment, waste handling and storage, pollution control, coastal reconstruction, protection and preservation, flood control, etc. Geosynthetics are of ever-growing importance in this field of applications. Geosynthetics are products made of synthetic or natural polymeric materials, which are used in contact with soil or rock and/or other geotechnical materials. The main basic classes of polymers used are polyethylene, polypropylene, polyester and polyvinyl chloride. However, within each class specific types of polymers (e.g. linear low density polyethylene octane- or hexane-copolymers, high-molecular weight polyethylene terephthalate with low concentration of carboxyl endgroups) and related additive packages (e.g. antioxidant packages) are selected, which fit best to the envisaged application. Clearly, the main field of application of geosynthetics is geotechnical and geoenvironmental engineering [1–3], even though they are applied in other fields of civil engineering, e.g. waterproofing of building constructions. The variety of products is impressive (figure 1) and the products are categorized as follows [4].

*Geotextile* (GTX): a planar, permeable, polymeric textile material, which may be nonwoven, knitted or woven. A nonwoven geotextile is a manufactured sheet, web or batt of directionally or randomly orientated fibers, filaments or other elements, mechanically and/or thermally and/or chemically bonded. A woven geotextile is produced by interlacing, usually at right angles, two or more sets of yarns, fibers, filaments, tapes or other elements. *Geogrid* (GGR): a planar, polymeric structure consisting of a regular open network of integrally connected tensile elements, which may be linked by extrusion, bonding or interlacing, whose openings are larger than the constituents are. Geogrids are used primarily for reinforcement applications. *Geonet* (GNT): a planar, polymeric structure consisting of a regular dense network, whose constituent elements are linked by knots or extrusion and whose openings are much larger than the constituents are. *Geomembrane* (GMB): a planar, relatively impermeable, polymeric sheet. *Geosynthetic clay liner* (GCL): an assembled structure of geosynthetic materials and low hydraulic conductivity soil material (clay) in the form of a manufactured sheet. *Geofoam* (GFO): a polymeric material, which has been formed by the application of the polymer in semi-liquid form, through the use of a foaming agent, which results in a lightweight material with high void content. *Geocomposite* (GCO): a manufactured or assembled material using at least one geosynthetic product among other components. An important example is the *geocomposite drain* (GCD): a prefabricated subsurface drainage product, which consists of a filter geotextile, a carrier geotextile and a polymeric drain core in between consisting of a geonet or another type of spacer.

The materials of the Earth’s crust are divided into two categories, soil and rock, and from the viewpoint of the geotechnical engineer the categories can roughly be distinguished by a simple experiment [5]. Soil is a natural aggregate of mineral grains that can be separated by such gentle mechanical means as agitation in water. Rock is a natural aggregate, which is connected by a strong and permanently acting cohesive force. Water can only destroy it by long-lasting erosion processes. Already from this description, it becomes apparent that the interaction between water and soil is of fundamental importance for geotechnical engineering. Water is pervasive. Soil properties strongly depend on the water content. Water intrusion can readily destroy earthwork constructions [5]. Another challenge for the geotechnical engineer is the low intrinsic tensile strength and elongation at break of soil materials [6]. Indeed, most of the functions of geosynthetics, which are described in this review, are dealing with the interaction of soil and water and the consequences of the disadvantages of the specific mechanical properties of soils.

With regard to geotechnical engineering, the function of the geosynthetics may be described more precisely as follows [4]. They are used as: (1) barrier, to prevent the migration of liquids or gases. (2) Containment, to contain soil or sediments to a specific geometry and prevent their loss. The contained fill takes the shape of the inflated at-rest geometry of the geosynthetic container. (3) Drainage layers, to collect and transport fluids. (4) Filter layers, to allow passage of fluids from a soil while preventing the uncontrolled passage of soil particles. (5) Protection layers, to prevent or reduce as a localized stress reduction layer the damage to a given surface or layer. (6) Reinforcement, to resist stresses or contain deformations in geotechnical structures. (7) Separation layer, to separate two dissimilar geotechnical materials to prevent intermixing. (8) Surficial erosion controller, to prevent the surface erosion of soil particles due to surface water run-off and/or wind forces. (9) Frictional interlayer, which is a layer introduced within an interface with the purpose of increasing or reducing friction across the interface. These functions can be fulfilled sustainably with respect to production and transportation, easily with respect to handling and installation as well as cost efficiently by the appropriately designed geosynthetic products. These are the reasons for the already very large and still growing market of geosynthetics.

**Figure 2.**
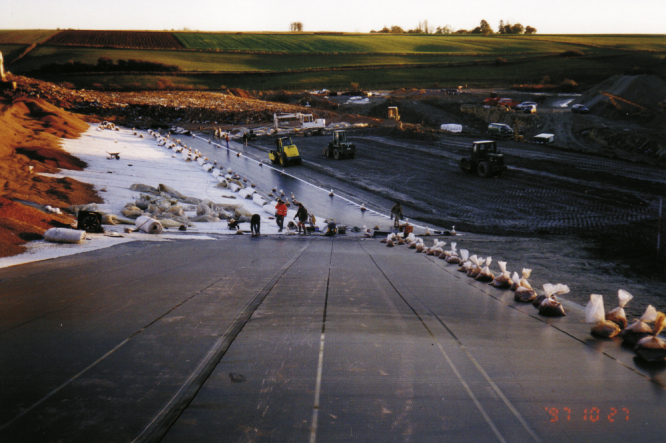
Geomembrane installation as part of the construction of a base liner system of a landfill. Source: Schicketanz, Aachen.

We focus on landfill lining, coastal protection and coastal hydraulic engineering, which are two important fields of application in geoenvironmental engineering. The importance may be seen for example by considering the number of papers related to these two topics submitted to conferences or published in journals. Two examples of the material problems related to the use of geosynthetics in landfill constructions will be discussed in detail: the effect of creep on the long-term performance of geocomposite drains and the numerical simulation of the interaction of soil with geogrids [7–10]. Both issues are of importance for the use of these products in landfill barrier systems (figure 2). With these two examples an issue is illustrated, which is of general importance for the use of advanced materials. A design engineer has to know in detail the specific characteristic of the material properties. Only in this case, reliable constructions can be designed and the materials can prove their advantages.

Various case studies are presented with respect to coastal protection and hydraulic engineering. The global warming leads, among many other effects of the related climate change, to a continuous sea level rise. According to the United States National Ocean Service, sea level has steadily risen since about the year 1900 and various measurement methods indicate a current rate of increase of 0.12 inches per year (corresponding to 30 cm in 100 years), which is due to the warming and related expansion of the ocean water body and the melting of land based ice. Regional differences are large, for example, an increase of 60 cm is expected until the end of the 21th century for the Baltic Sea. Storms and floods in connection with the sea level rise will have a potentially large and disastrous impact on island and coastal areas and the population living there. Therefore, the protection and reconstruction of coastlines is another issue of paramount environmental importance. In the case of coastal protection and reconstruction, the following impacts, which continuously threaten, alter and destroy coastal structures, have to be taken into consideration: strong forces due to waves and currents, loads due to seepage flows as well as the impact of erosion and sedimentation effects [11]. Finding solution to coastal and hydraulic problems related to these impacts was in Germany the starting point of the technical development and engineering applications of woven and nonwoven geotextiles [11–13]. Geotextiles are directly used or are applied in the form of composite construction elements, which are composed of a geotextile package filled with sand or other materials. The products are used as filter layers in dike and dam revetments as well as bed protection for dike floodgates and storm tide barrages, separation and filter layers at the foundation level of groynes and breakwaters, structural elements in the form of sand-filled tubes and bags, flexible bed stabilization mattresses for offshore and coastal protection structures and so on. Sandmats can effectively replace the former underwater technique, which applied fascines mattresses fabricated from willow saplings for bottom scour protection and bed stabilization at offshore- and coastal structures. The various functions, which geosynthetics may fulfill in the protection and reconstruction of coastal lines, are illustrated by the presented case studies [11].

Finally, we will consider the geosynthetic market and the economic benefits related to the use of geosynthetics. In addition, the positive ecological side effects (catchwords: carbon footprint and cumulated energy demand) of the use of geosynthetics are evaluated and some environmental concerns related to these materials are discussed.

## 2. Material problems related to geosynthetics: the performance of geocomposite drains in the long run [8]

**Figure 3.**
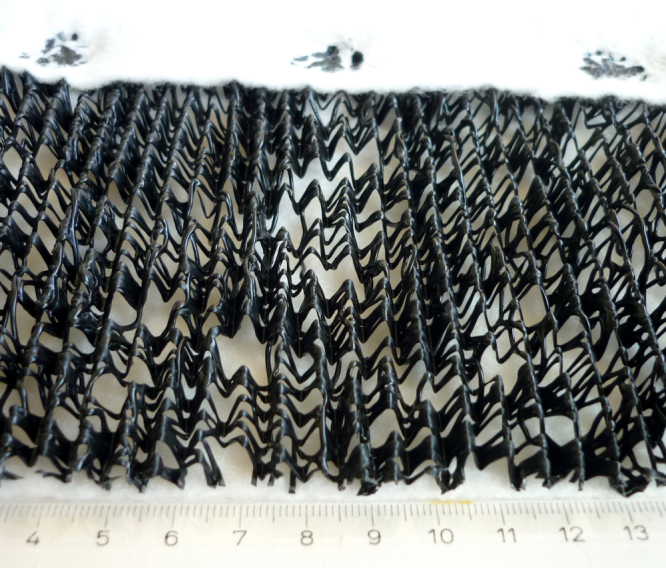
Geocomposite drain consisting of needle-punched nonwoven filter and carrier geotextiles of polypropylene staple fibers each having a mass per area of 200 g m^−^^2^ and a drain core of flexible wave shaped random arrays of extruded PP strands having a mass per area of about 600–700 g m^−^^2^.

**Figure 4.**
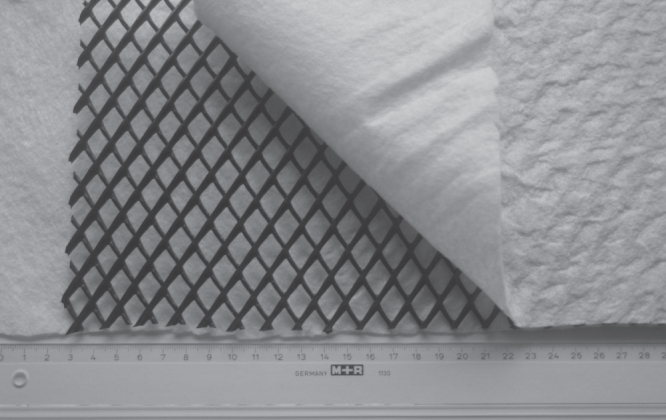
Geocomposite drain consisting of needle-punched nonwoven filter and carrier geotextiles of polypropylene staple fibers each having a mass per area of 200 g m^−^^2^. A biplanar HDPE geonet with mass per area of about 900 g m^−^^2^ was used as drain core.

**Figure 5.**
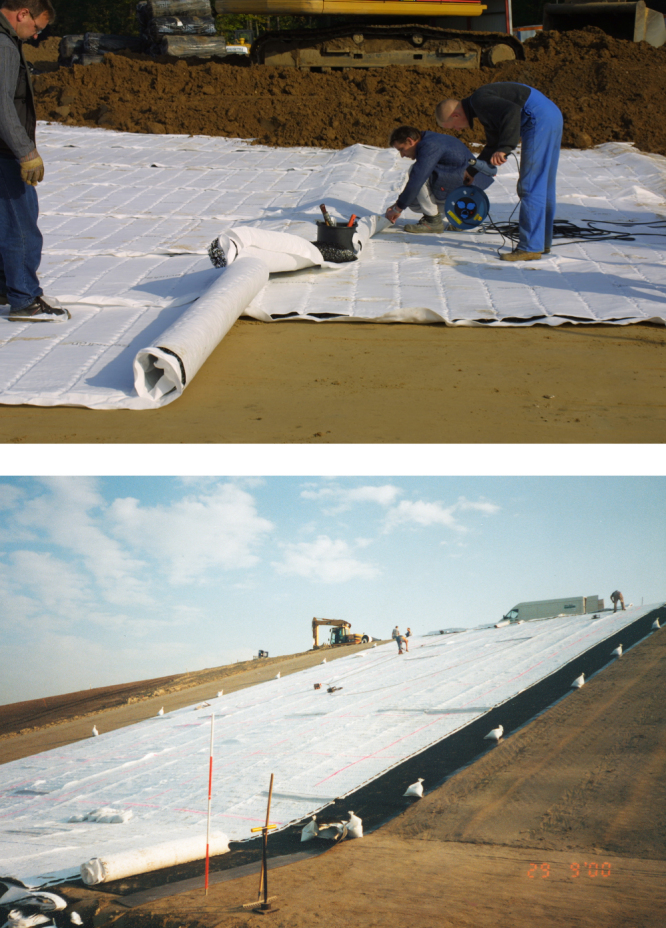
Installation of a geocomposite drain (top). Geocomposite drains are in most cases used on more or less steep slopes of landfill capping systems (bottom).

A geocomposite drain is made of three components: a filter geotextile, a drain core and a carrier/separation geotextile, as it is shown in figures 3 and 4 [1]. All three components are usually connected by some bonding techniques, e.g. welding, laminating or sewing. Under field conditions, the geocomposite drain is permanently stressed by a combined action of pressure and shear forces. An important example is the use of geocomposite drains on the more or less long and steep slopes of landfill capping system (figure 5), where the product is covered by a thick reclamation layer. Since the components are made of polymeric materials, the geocomposite drain and especially the open structure of its drain core will ‘creep’ and slowly and continuously be deformed under the applied forces. This creep deformation has a strong effect on the drainage capacity and the drain core stability in the long run. There are two types of behavior [7, 14]. First, in case of a very flexible drain core, the creep will lead to a continuous thickness reduction of the drain core structure and thereby to a continuous reduction in drainage capacity. Second, in case of a stiff and rigid drain core, the reduction in thickness due to creep will in fact be small. However, a stability failure may occur in the long run even though the stresses, which are induced by the field conditions, are much lower than the failure stresses, which were observed in a short-term compression experiment. Therefore, the drain core may collapse in the field in the long run, in which case it will lose most of its water flow capacity. The same reasoning about the effect of creep applies to the shear strength [15]. If the in-plane deformation due to creep becomes larger than a certain critical deformation, shear failure might occur in the field at a stress level which is significantly smaller than the one which was measured with a shear box equipment (direct shear test). In the following, we will show in which manner these long-term effects can be characterized by combining the results of creep experiments, long-term rupture tests under normal and shear stress, measurements of water flow capacity at high pressure as well as shear box tests. The testing institutes may use standard test methods to measure these ‘input’ properties [16]. Water flow capacity is measured according to ISO 12958 and internal shear strength by direct shear tests according to EN ISO 12957-1. Creep and creep rupture due to shear and normal stress is studied according to EN ISO 25619-1. In the long run, aging will significantly influence failure by inducing brittle rupture. Aging effects are very important. However, the phenomena exemplified in this review are solely due to creep and ductile creep rupture of pristine samples. Information about aging effects may be obtained elsewhere [17].

### 2.1. Long-term water flow capacity

**Figure 6.**
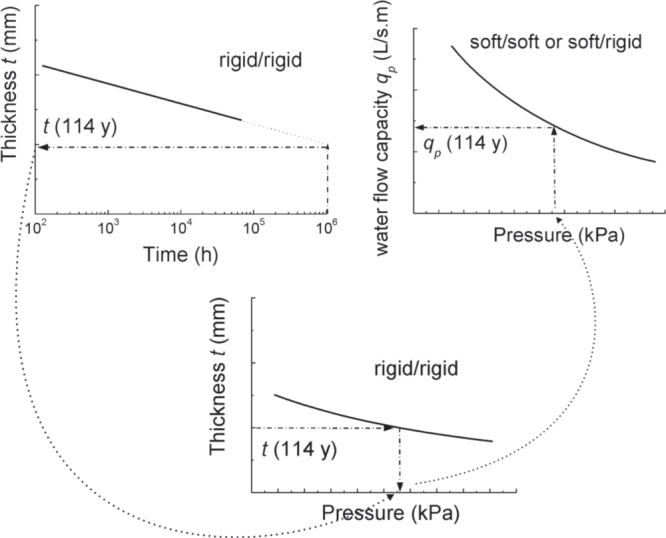
Schematic illustration of the method to determine the long-term water flow capacity of geocomposite drains from short-term experimental values.

**Figure 7.**
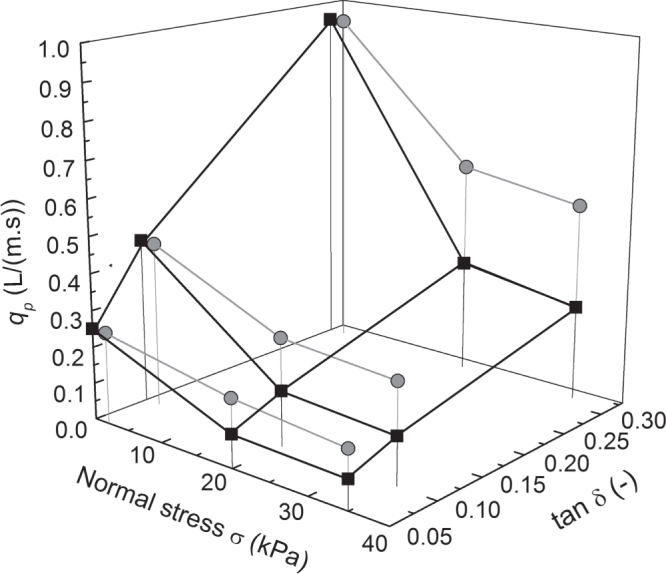
Short-term water flow capacity (gray) and long-term water flow capacity (black) as function of normal stress *σ* and tangent of slope angle *δ* (or ratio between shear and normal stress) for a bedding rigid/soft of a geocomposite drain sample as shown in figure 3.

Drainage capacity or water flow capacity *q*_*p*_ within the plane is defined as the volume of water, which can flow through the cross section of a geocomposite drain per unit width and per unit of time. It depends on the properties of the geocomposite drain, especially its thickness *t*, and on the hydraulic gradient *i* enforcing the flow in the plane of the geocomposite drain as well as on the so-called bedding, i.e. the confining properties of the layer above and below the geocomposite drain. Long-term water flow capacity may be determined as described in figure 6 [7, 18]. One starts by measuring creep curves related to various realistic loading conditions (*σ*_1_, *τ*_1_), (*σ*_2_, *τ*_2_), and so forth, where *σ* is the normal stress and *τ* is the shear stress. The residual thicknesses *t*_1_(114y), *t*_2_(114y), etc are extrapolated for the relevant design life (e.g. 10^6^ h or 114 years). In the second step, the water flow capacity is measured with a bedding condition rigid/rigid as function of pressure *p* applied to the test specimen. The rigid/rigid bedding condition allows simultaneously the monitoring of the thickness change. Thereby, the thickness as function of pressure is likewise obtained. Then, the pressures *p*_1_, *p*_2_, etc, which correspond to the respective residual thicknesses, are read from the pressure versus thickness curve. Imposing these pressures, the water flow capacities are determined for the bedding rigid/soft and soft/soft and the relevant hydraulic gradients. The values obtained are taken as long-term water flow capacities *q*_*p*_(114y) relevant for design at the various loading conditions (*σ*_1_, *τ*_1_), (*σ*_2_, *τ*_2_), etc. With respect to landfill capping design, one may finely construct a diagram (figure 7), which gives the relevant long-term water flow capacity as function of normal stress in the field and slope angle. These long-term values may be quite different from the experimental values of the declaration of performance associated with the CE-marking of the products or of other technical data sheets. The difference will depend strongly on the type of product. With the method described, the introduction of an arbitrarily chosen general reduction factor for creep in the construction design is avoided. However, there is an important problem. One measures a creep curve for, say, about one or two years, and one extrapolates to 100 years. During the 99 or 98 years, over which one extrapolates, things can happen, which could render the extrapolation senseless. This leads to the question of long-term shear strength and drain core stability.

### 2.2. Long-term shear strength

**Figure 8.**
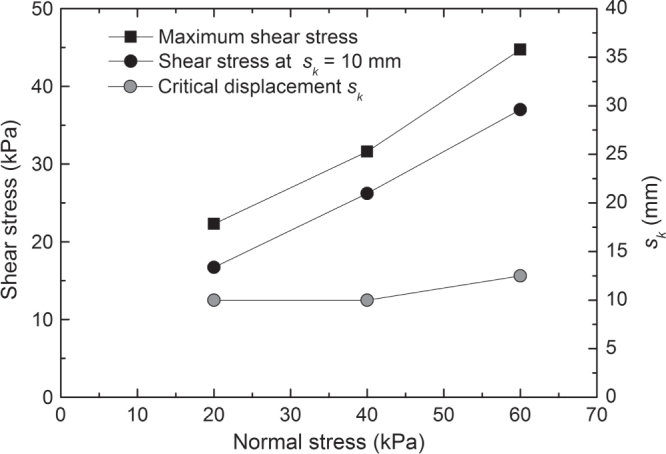
Shear strength envelope in the diagram of shear stress versus normal stress for a sample geocomposite drain as shown in figure 3 with welded components and critical displacement *s*_*k*_ as function of normal stress as obtained from a direct shear test.

**Figure 9.**
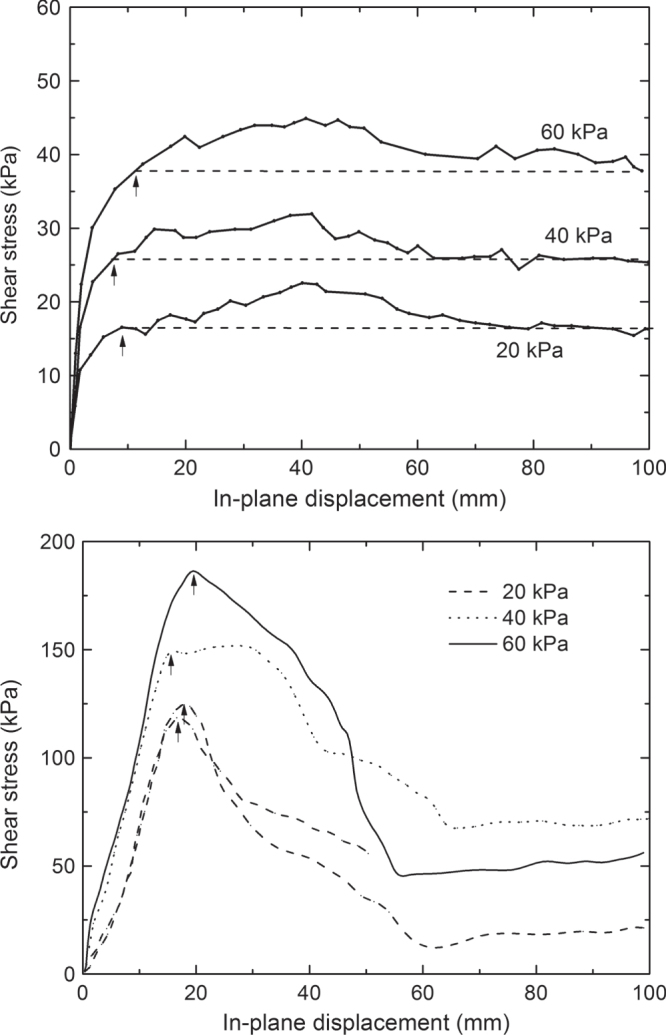
Progression of shear stress with the displacement at various normal stresses *σ* as typically observed in the direct shear test (geocomposite drain as in figure 3 with welded bonds (top) and as in figure 4 with laminated bonds (bottom)). Arrows mark the critical displacement *s*_*k*_ at which shear failure starts.

The components of the geocomposite drain can be solely connected to achieve easy handling during transportation and installation, e.g. by sewing them together. Internal shear strength of the geocomposite drain is then given by the friction between the components: The envelope of Mohr’s failure circles in the diagram of shear stress *τ* versus normal stress *σ* is nearly a straight line going through the origin, i.e. *τ* = tan(*ϕ*)*·σ*; where *ϕ* is the friction angle between the drain core and the geotextile. In many cases, the components are permanently bonded, e.g. by welding, laminating, etc, with the purpose to enhance significantly the shear strength. Then, the bonding enhances not only the angle *ϕ*. A large adhesion stress *a* is additionally observed in the direct shear test. The failure envelope obtained in the direct shear test is given by *τ* = tan(*ϕ*)*·σ* + *a*, as is shown in figure 8. However, bonds between the components are susceptible to creep. The creep deformation might cause shear failure at a combination of shear stress and normal stress in the field well below the stress level, which was derived from the failure envelope of the direct shear test. In other words, the stresses at shear failure, which are observed in direct shear testing with its more or less high deformation velocity, are usually significantly higher than those actually relevant to the conditions in the field, where the deformation velocities is smaller by orders of magnitude. Therefore, direct shear test results are not applicable to a proper design. However, a conservative estimate of the relevant stress at shear failure under field conditions might be obtained as follows. Figure 9 shows the development of the shear stress with in-plane displacement as it was observed in the direct shear tests of the two different types of products shown in figures 3 and 4. In the first case, yielding of the nonwoven geotextile at the welding points took place at a critical deformation *s*_*k*_. This was the relevant failure mode, even though shear stress continued to increase slightly to a maximum value at a larger deformation. In the second case, a sudden rupture occurred at the maximum level of shear stress and at a critical deformation *s*_*k*_. Considering the results of various other direct shear tests, we found that a ‘critical’ deformation *s*_*k*_ is associated with the relevant failure mode in many cases. It is quite independent from the applied normal stress and a characteristic feature of the product. We may now argue that as long as the creep deformation is below this critical deformation, there will be no shear failure in the long run. The creep curves under realistic loading conditions (*σ*, *τ*) may be analyzed to determine the in-plane deformation *s*_*c*_ associated with an expected service life *t*_*L*_. If *s*_*c*_ < *s*_*k*_, the loading (*σ*, *τ*) may be applied and no shear failure due to creep should occur within the life span *t*_*L*_. In case of the geocomposite drain shown in figure 3, the creep curve related to the loading condition *τ* = 11.7 kPa and *σ* = 35 kPa gave an extrapolated creep deformation *s*_*c*_ = 4 mm for a lifetime *t*_*L*_ = 100 y. This has to be compared with the ‘critical deformation *s*_*k*_, which is about 10 mm as indicated by the arrows in figure 9, upper part. In case of the geocomposite drain shown in figure 4, the creep curve related to the same loading condition gave an extrapolated creep deformation *s*_*c*_ = 2 mm for a lifetime *t*_*L*_ = 100 y. This has to be compared with *s*_*k*_ = 17 mm (see arrows in figure 9, lower part). In both cases, the connection between the components of the GCL may be considered as quite strong and only weakly affected by creep for the given loading conditions.

### 2.3. Drain core stability

Collapse of the drain core and shear rupture are independent phenomena. One may have a collapse of the drain core without any effect on the shear strength and one may have shear failure even though the drain core remains completely intact. In the long-term creep rupture test at high pressures, the failure behavior is usually dominated by drain core collapse. The line of reasoning as described in section 2.2 can be applied to the problem of drain core stability, if a critical thickness can be identified from long-term creep tests [7]. However, the problem of drain core stability might be directly solved by the measurement of failure time with respect to drain core collapse as function of the loading condition (*σ*, *τ*) and by the extrapolation of critical loading conditions related to lifetime from a semi-logarithmic plot of failure time versus loading conditions.

**Figure 10.**
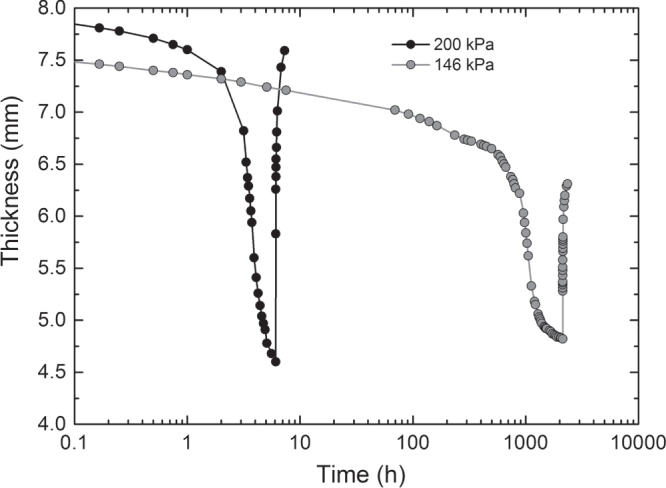
Creep curves as obtained for the geocomposite drain product shown in figure 3 at normal stresses of 146 and 200 kPa. The polymeric drain core recovers from collapse after releasing the stress.

**Figure 11.**
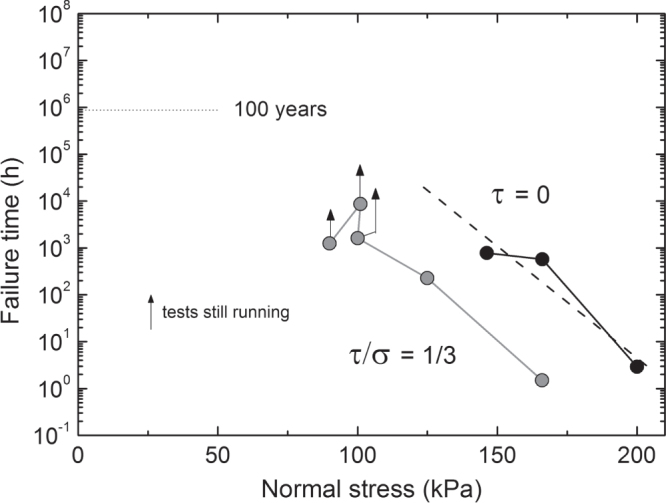
Failure time with respect to drain core collapse versus normal stress as measured in creep rupture tests of the geocomposite drain sample shown in figure 3. Gray circles: ratio of shear stress to normal stress 1:3. Black circles: pure normal stress.

Figure 10 shows results of a long-term creep test following EN ISO 25619-1 at very high normal stress. After a certain failure time, the drain core collapsed. Decreasing normal stress the failure time increased significantly. Figure 11 shows results of such tests from an ongoing testing project. Superposing a shear stress in addition to the normal stress will decrease the failure time. Therefore, the tests were performed at a given ratio of shear stress to normal stress and the failure times are measured for varying normal stresses. In figure 11 the logarithm of failure time is plotted as function of normal stress for *τ*:*σ* = 1:3 and pure normal stress *σ* [7, 8]. With enough data available, the critical normal stress related to an envisaged service life, above which collapse might be imminent, can be extrapolated by a best-fit straight line. The critical normal stress is the abscissa of the intersection of the best-fit straight line with the horizontal line indicating the envisaged lifetime. As one can imagine by considering the data in figure 11, drain core stability is an issue of real concern in case of severe loading conditions and very long expected lifetimes.

### 2.4. Design considerations

The use of geocomposite drains in capping systems of landfills is a typical example were loading conditions are severe and envisaged lifetime is very long. With thick reclamation layers on top of the geocomposite drain, normal stresses of about 20 to 40 kN m^−^^2^ are obtained. The tangent of the slope angle *δ* of a waste pile and therefore the respective ratios of shear stress to normal stress, *τ*/*σ* = tan *δ*, are typically in the range of 1V:3H to 1V:2H. Design life has to be many decades, actually at least 100 years according to the German regulations [19]. To design the drainage layer reliably, it is very important to know the long-term water flow capacity as function of the loading conditions and the critical normal stress and the critical ratio of shear stress to normal stress for a geocomposite drain product. In sections 2.1–2.3, it was discussed how these characteristic properties of a product can be derived using standardized test procedures. However, various other impacts may affect the performance of the geocomposite drain. For an appropriate design the relevant design values of the water flow capacity have to be calculated from the characteristic values of the long-term water flow capacity (see section 2.1 and figure 7) by using additional reduction factors. The reduction factors in table 1 are recommended by the working group 6.1 ‘Geotechnics of Landfills and Reclamation of Contaminated Sites’ of the German Geotechnical Society (see recommendation E2-20, www.gdaonline.de/empfehlung.html). They may be considered as the inverse of partial factors of safety with respect to the various impacts. They have been the result of a survey of the opinions of the experts of this group. The values are still under discussion. However, they are in the lower part of the ranges, which are given by other sources [1]. It is recommended by the working group to use an additional partial factor of safety for the uncertainty in assessing the inflow of rain water to the drainage system. If a maximum seepage of 25 mm d^−1^ (for Germany) is assumed this partial factor of safety can be set to 1. The overall global factor of safety obtained by multiplying the inverse of the reduction factors is about 2 with respect to the assumed water inflow and experimentally determined long term water flow capacity. Currently, there is a controversy in Germany, whether this factor is still too small for landfill capping systems, because the effects of root penetration might be underestimated in case of a reclamation layer, which is typically 1 m thick.

**Table 1.** Reduction factors RF. Long-term water flow capacity (see section 2.1) has to be divided by these factors to obtain the design value according to the recommendation E2-20 of the working group 6.1 of the German Geotechnical Society.

Symbol	Description	Value
RF_1_	Reduction factor for scattering of experimental data and systematic errors in estimating long-term behavior	1.3
RF_2_	Reduction factor for impairment of water flow capacity by stress and small damage due to construction work	≥1.2
RF_3_	Reduction factor for decrease in geocomposite drain cross section and water flow capacity due to overlap joints, butt joints or connections to components of the construction	1.2
RF_4_	Reduction factor for impairment of water flow capacity by precipitation, root penetration and infiltration of soil particles	1.1–2.0

According to figure 11, lifetime increases by reducing normal stress *σ* for a given ratio (*τ*/*σ*)_0_. Conversely, the line of best fit will be shifted to the right for decreasing ratio *τ*/*σ* and thereby lifetime increases for a given normal stress *σ*_0_. Therefore, the critical normal stress should be either multiplied by a factor of safety and the ratio of shear stress to normal stress taken as it is, or the ratio should be multiplied by a factor of safety and the critical normal stress may be applied as taken from the diagram. The design of landfill drainage layers is described in more detail in the above-mentioned recommendation E2-20 of the German Geotechnical Society.

## 3. Material problems related to geosynthetics: long-term pull-out resistance and material properties of geogrids [10]

**Figure 12.**
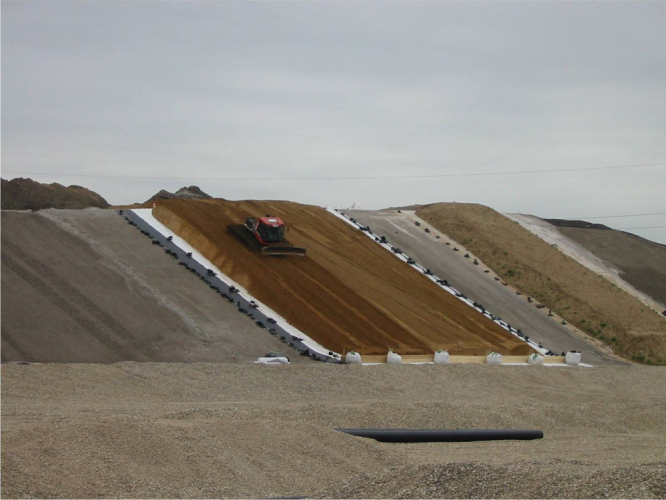
Geogrids are used to prevent sliding on long and steep slopes during installation and use of a landfill capping system [58].

**Figure 13.**
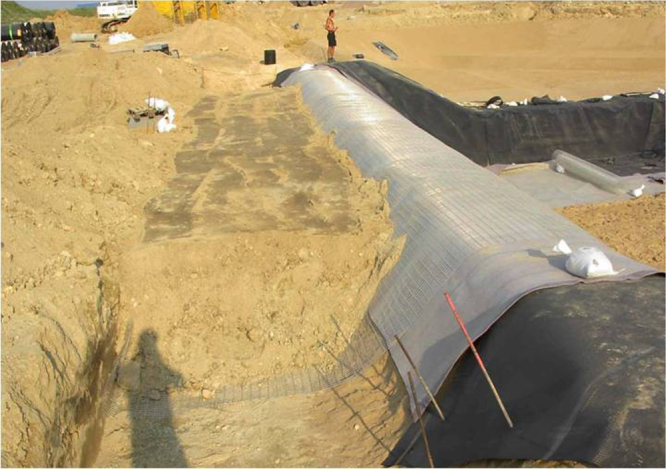
Anchorage trench of a geogrid [59].

**Figure 14.**
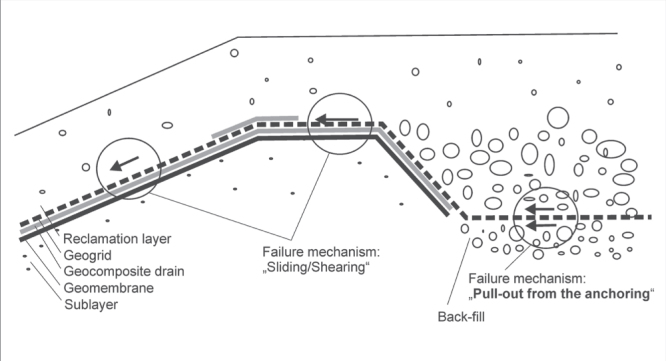
Schematic picture of an anchoring construction and the various components in a landfill capping system. The relevant failure mechanisms are indicated.

**Figure 15.**
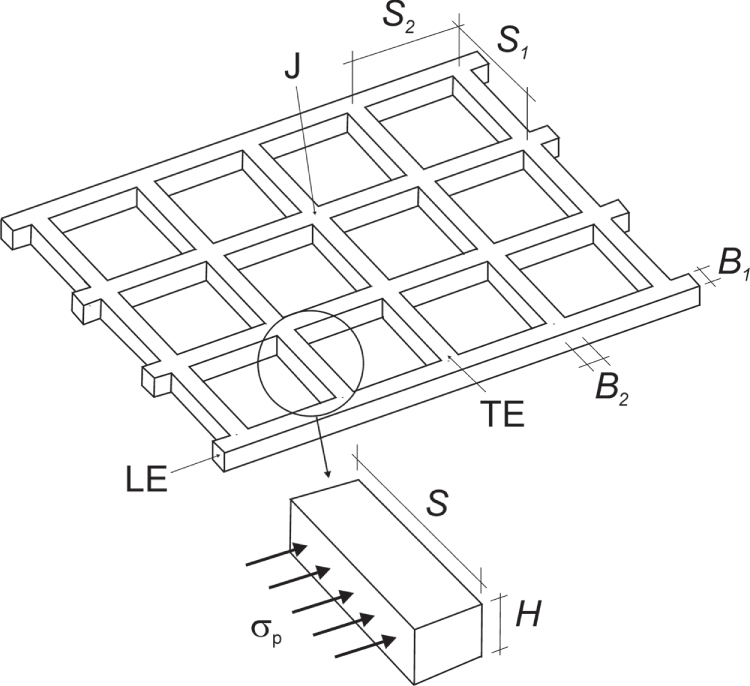
Schematic view of a geogrid. It consists of longitudinal elements (LE) and transverse elements (TE). At the crossing of both elements are the junctions (J). The indicated parameters are used to describe the geometry of the geogrid. The soil which surrounds the embedded geogrid and fills the openings will resist any movement of the geogrid. Thereby an ‘Earth pressure’ or a ‘bearing resistance’ *σ*_p_ is mobilized in front of each section of the TE.

In the following, the pull-out behavior of an idealized geogrid with limited junction strength is considered and some conclusions are drawn with respect to the material properties of geogrids, which are actually relevant for a safe design of the anchoring [9]. The focus is on the application of geogrids in landfill capping systems, where they are used to prevent sliding failure on long and steep slopes (figure 12). For such an application, it is important to construct an anchoring of the geogrid with high enough pull-out resistance (figures 13 and 14). A geogrid is composed of longitudinal elements (LEs), of transverse elements (TEs) and of junctions (J) (figure 15) [1, 20, 21]. The soil–geogrid interaction is due to friction between the soil particles and the surface of the geogrid [22]. In addition, there is a ‘soil resistance’ *σ*_p_ mobilized in front of the TEs [22]. The tensile force in the LEs is transferred via the junctions into the TEs and compensated by the mobilized ‘bearing force’ of the soil. If one assumes that the LEs are totally stiff and that the junctions have a strength, which is always much larger than the induced stresses at the junctions, one may derive a simple formula for the pull-out resistance: The pull-out force per unit with of a geogrid is proportional to the anchorage length and to the normal stress on the geogrid in the anchorage [22]. It follows that the pull-out resistance can be made arbitrarily large by increasing anchorage length or normal stress. Therefore, the tensile strength of the LE is usually considered as the only relevant material property of a geogrid [23]. However, the assumptions may apply to metal geogrids. They do not apply to polymeric geogrids.

**Figure 16.**
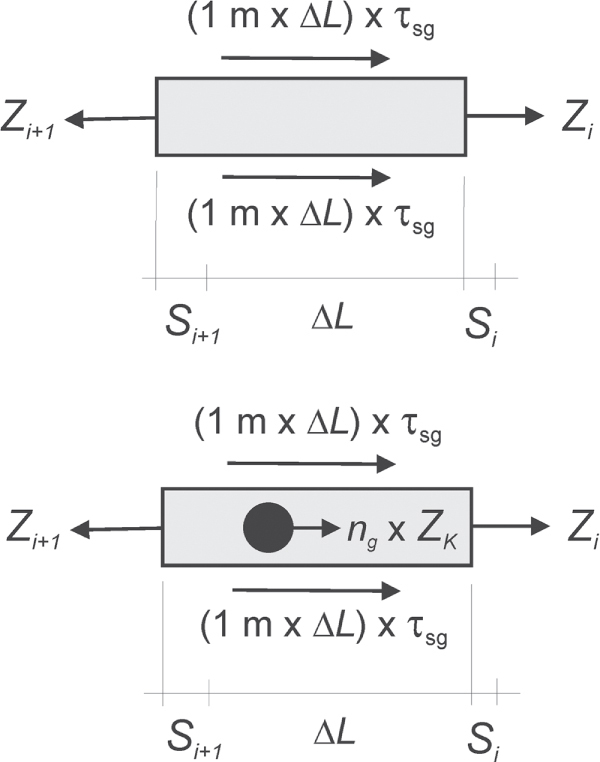
Illustration of the discrete segment model, which was used to simulate the load transfer mechanisms and pull-out behavior of a geogrid. Above: a normal segment of the longitudinal element, below: a segment, which contained a transverse element with *n*_*g*_ junctions.

A better understanding of the soil–geogrid-interaction may be obtained from numerical simulations. In the following, a numerical simulation is discussed which applies the so-called discrete segment model as presented in [24, 25]. A different version of the model was suggested in [26]. Our discussion refers to the consideration given in [25–28]. Consider a geogrid in the anchorage (figure 14). The geogrid is divided along the anchorage length *L* into small segments of length *ΔL* and unit width (1 m). Some segments of the LE contain a TE and the number of junctions associated with this segment is given by the number of junction *n*_*g*_ in the TE per unit width (figure 16). The segments are consecutively numbered starting with zero at the end of the anchorage and going to *n* at the front of the anchorage. Therefore, *L* = *n* × *ΔL.* Segment *i* is pulled by segment *I* + 1 with *Z*_*i*+1_ = tensile force per unit width (1 m), and it pulls segment *i* − 1 with *Z*_*i*_. Within each segment, part of the tensile force is transferred into the soil via surface friction between soil particles and the geogrid surface. In case of segments with TE, there is, in addition, a transfer of tensile force via the J and TE into the soil. It is compensated by the bearing force of the soil, which is mobilized in front of the TE (figure 15). The segments and forces are illustrated by figure 16. Let *τ*_sg_ = the shear stress due to surface friction and *Z*_*K*_ = the tensile force transferred per J into the bearing soil in front of the TE, then, the balance of forces is given by equation (1a) for segments without TE and equation (1b) for segments with TE. Note that *τ*_sg_ and *Z*_*K*_ are functions of the displacement *s*_*i*_ of the segment *i*

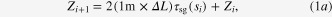


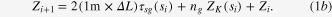


Displacement of the segments is induced by the lengthening due to the strain *∊* of each segment due to the tensile forces. Let *s*_*i*_ = the displacement of element *i* due to the displacements of all segments with number smaller than *i*. Segment *i* adds its own elongation and the displacement of segment *s*_*i*+1_ is therefore:

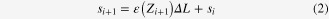

**Figure 17.**
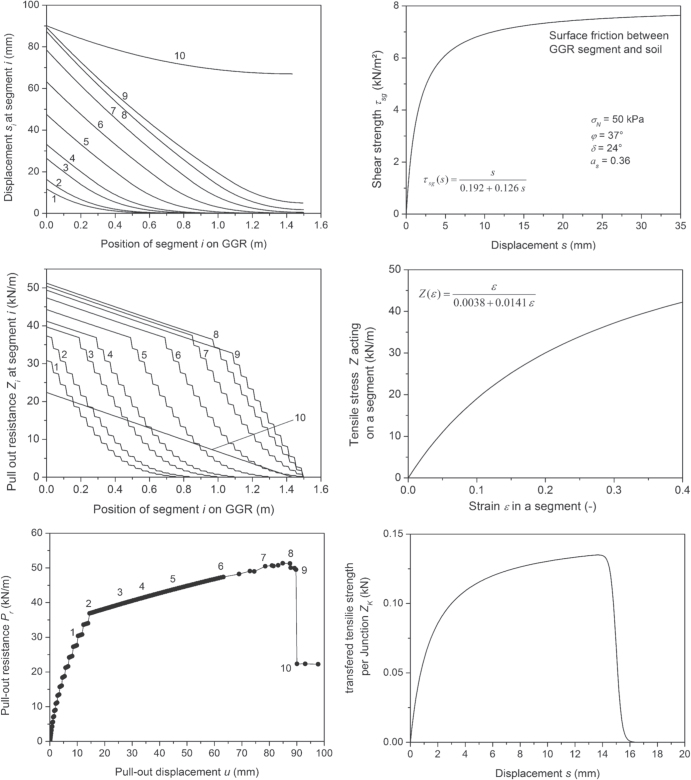
Example of the relation between shear strength *τ*_sg_(*s*) due to interface friction and displacement *s* at a segment (top-right panel). Such a type of curve is typically measured in a shear box test of the friction between geogrid and soil. Example of a diagram of tensile force Z versus strain *∊* used for a segment (middle-right panel). Tensile strength which is transfered per junction *Z*_*K*_(*s*) from LE to TE and compensated by the soil resistance as function of displacement *s* (bottom-right panel). Failure of the junction may occur at certain level. Distribution of the pull-out resistance (top-left panel) and the displacement (middle-left panel) along the imbedded geogrid length during a ‘numerical’ pull-out test of a flexible geogrid with limited junction strength. The result of the simulated pull-out test is shown at the bottom-left panel: pull-out resistance *P*_r_ of the geogrid versus pull-out displacement *u*. Various stages of the pull-out process are indicated by consecutive numbers.

*∊*(*Z*_*i*+1_) is the strain as function of the tensile force, which acts on a segment of basic length *ΔL*. *Z*_*i*+1_(*∊*) is the inverse function, i.e. it gives tensile force versus strain *∊*. The three functions *τ*_sg_(*s*_*i*_), *Z*_*i*+1_(*∊*) and *Z*_*K*_(*s*_*i*_) actually determine the behavior of a geosynthetic geogrid (see figure 17, right panel from top to bottom). The function *Z*_*i*+1_(*∊*) is primarily given by the materials properties of the LE. It may be derived from tensile or pull-out tests [25]. The other two functions describe the interaction between soil and geogrid. These functions depend on the following parameters: *a*_*s*_ = fraction of the unit of area, which amounts to the surface of the geogrid. 1−*a*_*s*_ = fraction of the unit area, which amounts to the openings of the geogrid. *ϕ* = friction angle of the soil. *δ* = angle of surface friction between soil and geogrid. *σ*_*N*_ = normal stress on the geogrid in the anchorage. Finally, the geometry of TE, grain size distribution of the soil and the associated bearing force are of relevance [22]. *τ*_sg_(*s*_*i*_) may be obtained from direct shear tests [22]. *Z*_*K*_(*s*_*i*_) might be obtained from special pull-out tests as well [21, 25–27]. The calculation finally delivers the pull-out resistance *P*_*r*_ = *Z*_*n*_ as well as the pull-out displacement *u* = *s*_*n*_, which would be found in a ‘numerical’ pull-out test with a geogrid behavior as described by the model.

The behavior of a so-called bearing force geogrid, i.e. a product, where not only friction but also bearing forces significantly contribute to pull-out resistance, is now considered with rupture of the J at a level below the mobilized bearing force. Therefore, a modified function *Z*_*K*_(*s*_*i*_) is introduced, which describes what happens at the J [26]: at a certain limit of J strength rupture occurs and the tensile force transferred via the junctions drops to zero. Figure 17 (right panel) shows the functions *Z*_*i*+1_(*∊*) and *τ*_sg_(*s*_*i*_) and the associated parameters (top and middle) and the new function *Z*_*K*_(*s*_*i*_) (below). Beside the cut-off in the function *Z*_*K*_(*s*_*i*_), which is only relevant for the behavior in the long run and usually not visible in a short-term pull-out test, these model functions are representative examples of functions which may be actually obtained from real pull-out tests with real geogrids. In the calculation, it was assumed that *ΔL* = 1 cm. Every fourth segment was one with a TE and the number *n*_*g*_ of J per unit width associated with this element was 20. Figure 17 (left panel) shows the results. It gives the displacement at different positions along the geogrid (top), the distribution of the pull-out resistance along the geogrid position (middle) and the diagram of pull-out resistance versus pull-out displacement of the hypothetical pull-out test (bottom). Different stages of the simulated pull-out behavior are indicated. According to these results, the pull-out process of a geogrid with limited junction strength may be described as follows.

Stage 1: with increasing pull-out force, an increasing part of the geogrid is strained and the deformation moves into the imbedded geogrid (figure 17, left panel, top). The steps in the pull-out resistance along the already strained and ‘activated’ length are due to the transfer of tensile load into the soil via J and TE (figure 17, left panel, middle). Displacement is large in front of the activated length and zero at the end. Therefore, the steps are high in front of the activated length. Their height decreases continuously along the activated part of the anchorage length.

Stage 2: a critical pull-out resistance is reached, because the most loaded J ruptures in front of the imbedded geogrid. Their mechanical strength is exceeded. Associated with the critical pull-out resistance is a critical activated anchorage length.

Stage 3, 4, 5 and 6: row after row of TE, the J are driven above their strength and rupture. Further increase in pull-out resistance is only due to surface friction. New rows of J are activated at the backmost area of the anchorage length. The range, where J are activated and rupture, moves along the geogrid as if driven by a zip-opener mechanism.

Stage 7: at this stage, the activation reaches the end of the embedded geogrid. The geogrid is not only elongated but the segment at the end and therefore the entire geogird is actually forced to move.

Stage 8: the J at the end of the geogrid are mobilized and the loading of the J over the whole activated length become more and more equal.

Stage 9: the mechanical strength is exceeded in all remaining J and they rupture, just after maximum pull-out resistance is reached. Because of failure of all the J, pull-out resistance collapses. The geogrid contracts by a certain amount of length and is pulled out by the same amount.

Stage 10: the geogrid is ‘caught’ by the surface friction on a much lower level of pull-out resistance.

In a real pull-out experiment the loading and deformation ‘history’ of the individual J in the course of the experiment will vary around the ‘ideal’ behavior as described in the ‘numerical’ experiment. The J will not accurately fail row after row at the same time. Ruptured J will be distributed over a certain area. Boundary effects will influence failure behavior in a real experiment. Therefore, an experimental curve of pull-out resistance versus displacement as obtained in a real experiment will only approximately fit to the theoretical one. The corners in the curve are rounded off and the collapse is less steep.

However, from the simulation one has to draw various conclusions with respect to geogrid behavior with limited junction strength and relevant material resistances of a polymeric geogrid. Pull-out resistance cannot simply be increased by increasing anchoring length and normal stress, because the LE are not totally stiff and the strength of the junction is limited. Assuming that all the junctions fail and that this failure does not impair the tensile properties of the LE, the pull-out resistance is only due to frictional resistance of the actually installed anchoring length. Depending on the type of geogrid, this ‘residual’ pull-out resistance may be significantly lower than the one expected according to the usually applied design rule. The bearing effect of the TE contributes substantially to the pull-out resistance [28]. However, this contribution may be limited by a limited junction strength. There is a critical anchoring length and a critical pull-out resistance, which is related to the failure of the junctions. To avoid failure of junctions, these critical values have to be taken into account. Therefore, junction strength is a material resistance, which is as important for a safe design as the long-term tensile strength of the LEs.

Envisaged lifetime of geogrids is often very long. In Germany, for example, the landfill regulations require the proof that the anchoring of a geogrid is safe for at least 100 years. Junction strength is affected by creep and aging in such a long period of time at least as large as the tensile strength of the LEs and TEs [29, 30]. Furthermore, no justification of the assumption is available that long-term junction strength is always much larger than the averaged bearing force per junction in the anchoring, even though laboratory values of short-term junction strength are usually significantly higher than this averaged bearing force [31, 32]. This applies to all kind of geogrids (welded, woven or extruded). It follows, that every polymeric geogrid has to be considered *ab initio* as a grid with limited long-term junction strength. The simulation shows that experimental studies of the aging behavior of junctions are a prerequisite for a safe design and that the design rules have to be modified to take limited junction strength explicitly into account [9].

## 4. An important field of application: hydraulic and coastal engineering [11]

**Figure 18.**
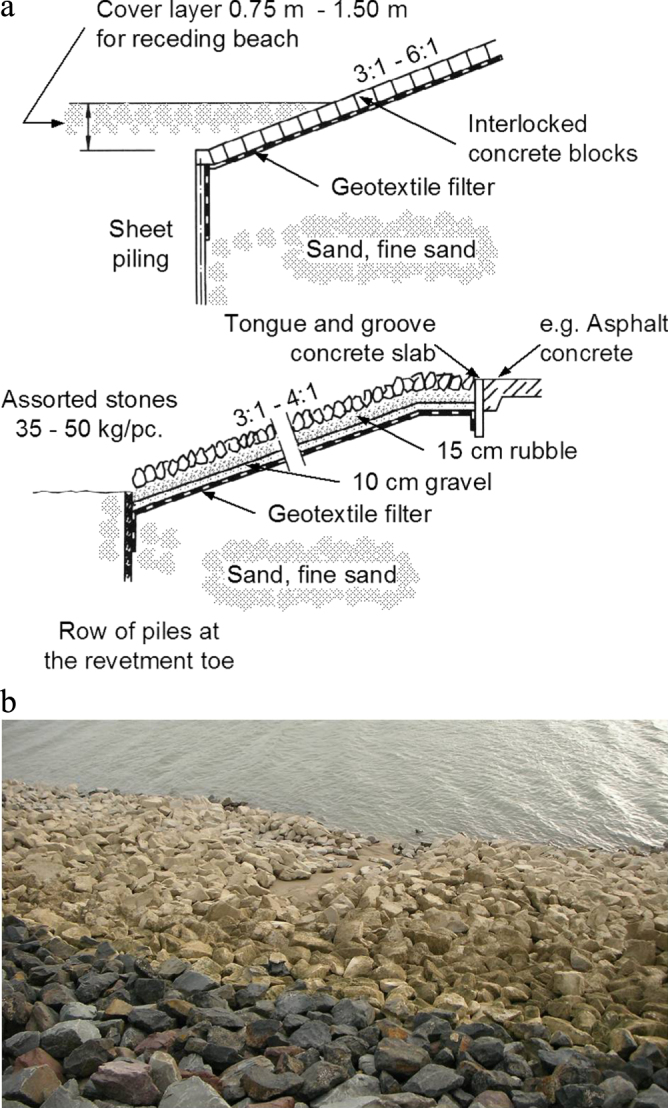
(a) Schematic view of revetments of dikes and forelands [11]. (b) Canal embankment: relocation of revetment elements and exposure of the layer underneath. Geotextile filters may prevent erosion of the mineral liner [33].

After having discussed the relevance of the specific material behavior of geosynthetics to the engineering and design of landfill constructions, we will now discuss the use of geotextiles in coastal engineering, which is intimately related to environmental problems. We will illustrate this field of application by shortly describing a few case studies. More details about side conditions and the performance of the geosynthetics in each case may be obtained from the cited literature. The ‘classical’ example of the use of geotextiles is their application to revetments, which protect foreland edges and toes of dikes and dams (figure 18(a)). These constructions are flown through by strong alternating currents due to tidal ebbs and floods as well as ship propeller wash. Therefore, a filter layer is necessary, which blocks the passage of fine soil material and prevents erosion, but which does not impede the escape of the water. Revetments with geotextile filters are far more resistant to wave impact than comparable revetments with a filter layer of mineral grains [33]. Geotextile filters are able to bridge areas, where the stone revetments were destroyed by propeller wash and therefore to successfully protect the clay liner underneath (figure 18(b)). This was shown, for example, in a study performed at the German Dortmund-Ems-Canal (DEC). The investigations were undertaken in 2005, when the DEC was accidentally drained on a length of 7.7 km due to a leakage caused by construction works, and in 2006, when the same section had to be emptied deliberately. Previous studies at the German Mittellandkanal had already indicated that a mineral filter is not able to protect the clay liner underneath if the lining on banks and beds is loaded by propeller wash. The investigations at the DEC thus focused on the comparison of sections built with geotextile and mineral filters, respectively. Sections, which were built under water, were compared to sections built in dry conditions, too. In most parts of the sections with high ship loading the embankment protection was in a poor state. Damages, such as erosion and relocation of revetments elements as well as formation of water channels, upheaval and the exposure of the filter were detected (figure 18(b)). In sections with a geotextile filter, the mineral sealing liner underneath the filter was usually still intact at these ‘bad spots’, while mineral filters were often washed away leaving the sealing liner unprotected. Partly, even liner erosion had already started. Both, the revetments built under water and in dry state showed comparable damages. Thus, it may be concluded that most damages were actually caused by ships. After the relocation of the revetment and erosion of the filter, the stability of the waterside embankment is no longer guaranteed and the stability of the whole dams is in danger. With respect to the safety of the construction, it follows, that particularly for high dam sections a geotextile filter should become the standard choice, because it provides erosion protection in case of local exposure of the filter and sealing layer while a mineral filter does not. In coastal engineering geotextile filters are also used for erosion and scour protection, which is necessary at waterside revetments and groyne toes. They provide a large flexibility in the construction designs.

Geosynthetic are an important part of modern dike constructions [34]. If marl or clay is not available for covering the sand core of a dike the use of a geotextile as an erosion-resistant encapsulation material for the sand core offers a technically appealing solution. Instead of mineral clay, a geosynthetic clay liner can be installed as seaside dike core coverage. Slightly loaded dikes with a sand core encapsulated by a geotextile may be protected using concrete paving stones or a topsoil cover layer. If a sufficiently robust geotextile is installed as a filter, horizontally and vertically interlocking concrete paving stones may be laid on the outer embankment without the need for an additional protection layer. If the slope of the outer embankment is very gentle and wave loading is slight, a geotextile may be installed as a cover layer for a sand-core dike. A layer of topsoil with a minimum thickness of 20 cm may be applied to provide a protection layer as well as a cultivation bed for vegetation.

**Figure 19.**
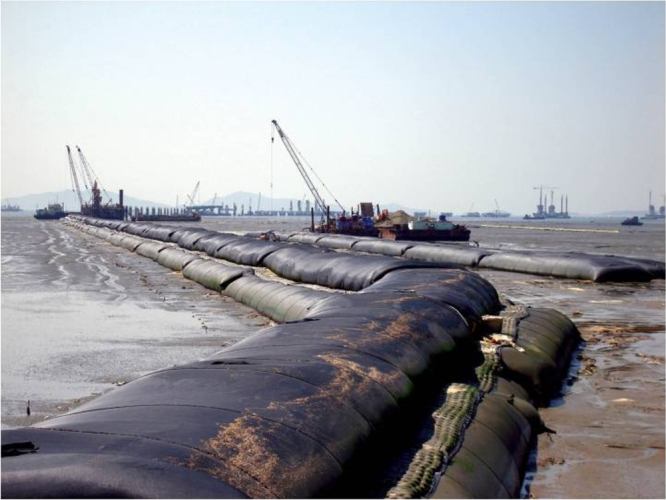
Geotextile tubes hyraulically filled with sand as reclamation dike unit. Source: [60].

**Figure 20.**
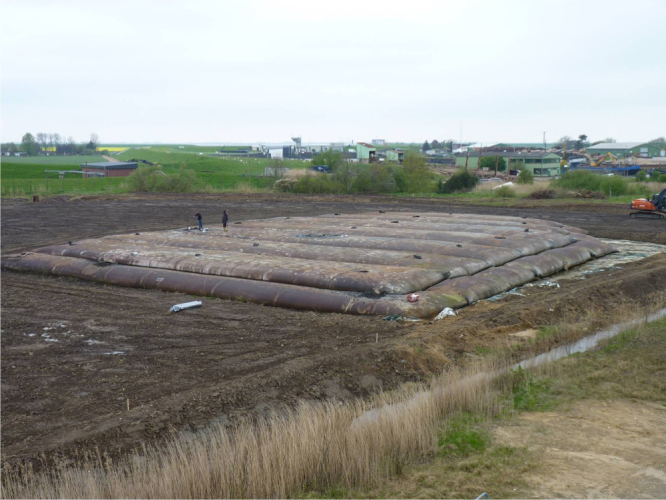
Test field of geotextile tubes hyraulically filled with sand for scour protection. Source: Cantré and Saathoff, Universität Rostock, Chair of Geotechnics and Coastal Engineering.

**Figure 21.**
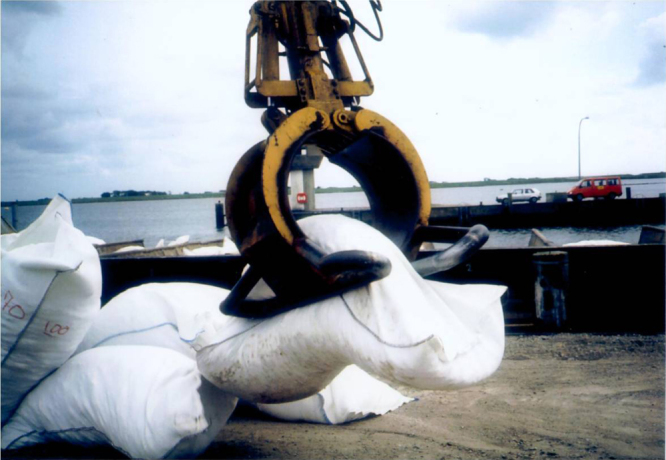
Nonwoven geotextile containers have advantages compared to woven geotextile containers because of high robustness and large elongation at break [36, 37].

The last one and a half decades have shown a large growth in the application of geotextile container technology with highly visible and spectacular projects. The scale of the containers ranges over about two orders of magnitude from small bags, over mats to large tubes with 2–5 m of diameter and tens of meters of length (figures 19 and 20). The deployment of sand-filled bags and tubes in coastal protection applications permits the use of locally available dredged sand for producing structural elements or entire structures. The normal size of sand bags for the temporary repair of damaged dikes is 40 × 70 cm, with a volumetric capacity of up to 1 m^3^. Hand sacks can be stored in filled condition so that they can immediately be used in emergency. The geotextile containers are sewn, tied up or closed with double-sided clinging strips after they have been filled. The filled containers can easily be handled and have a high static friction when they are piled up. On the other end of the size scale are very large sand filled tubes. For example, in case of the Saemangeum Seawall project in Korea, which is considered as the ‘world largest sea dike’, the basic underwater berms of the sea dike were constructed by a stack of geotextile tubes instead of a rock-fill [35]. In the original design, the first layer of sand-fill of the polder dike was constructed between two rock-fill berms, which retained the sand-fill core up to the water level. The second layer of sand-fill was constructed on top of the first layer with exposed gentle side slopes. As an alternative design, a stack of large geotextile tubes were used instead of the rock-fill berm in one of the construction packages. The diameter of the geotextile tubes ranged from 2 to 4 m and the circumference from 6.3 to 12.6 m. The height of the filled tube was between 1.1 and 2.2 m and the volume filled with dredged sand was between 2.2 and 9.4 m^3^ per unit of tube length. More than 26 km of geotextile tubes were used for this project. Nonwoven fabric containers with sufficient strength are usually best suited to fulfill the various design requirements (figure 21). They have a high flexibility and good friction properties. Especially at dynamic sandy beaches, where the use of rocks, steel and concrete as ‘hard coastal structures’ is contrary to the ‘soft coastal protection’ philosophy, geotextile sand filled containers made of needle-punched nonwovens as ‘soft rock structures’ can lead to new flexible technical solutions which are often better than the conventional one due to adaptability of the geocontainer construction to cyclical hydrodynamic loads and morphological changes resulting from the long-term and short-term fluctuations of the sea bed.

### 4.1. Case study: stabilizing the scour embankments at the Eider river storm tide barrage [36, 37]

**Figure 22.**
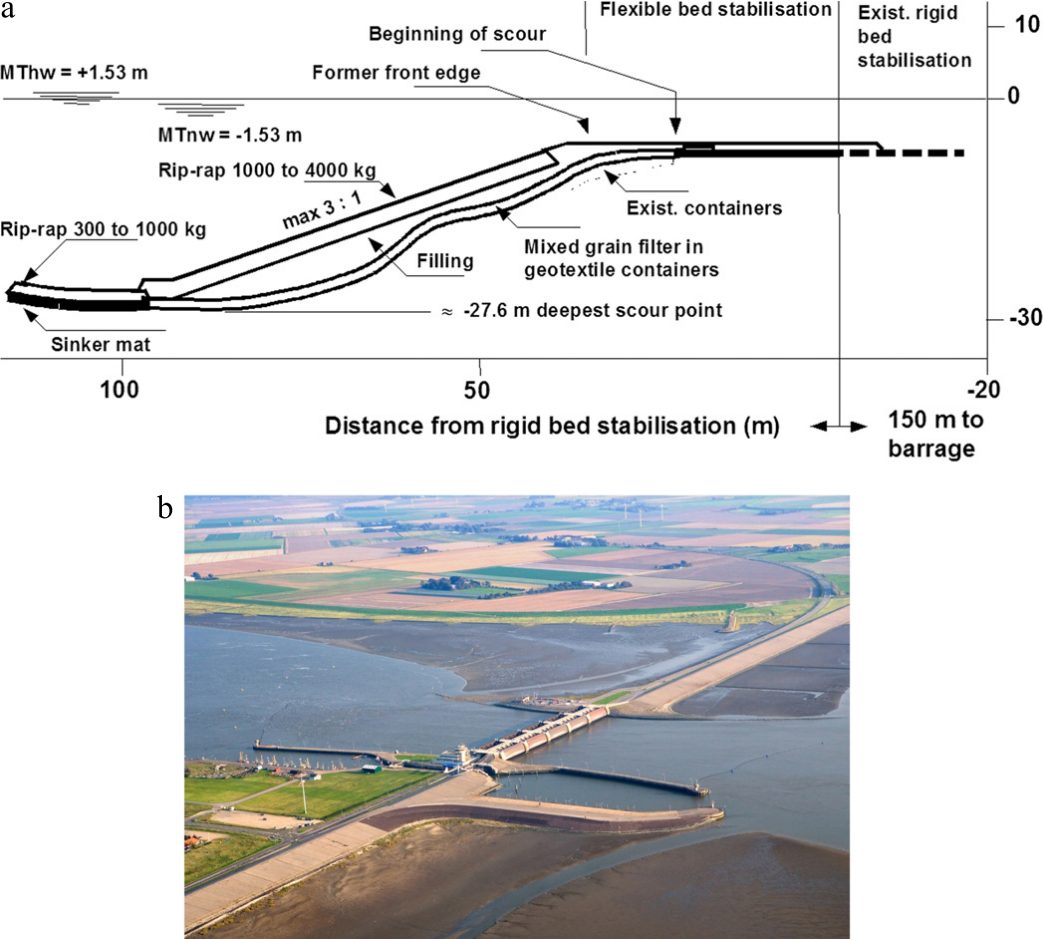
(a) Storm tide barrage at the Eider river. Scheme of the realized profile of bed stabilization at the outer embankment [36, 37]. It shows the crossover from the deep, seaward scour (left) to the shallow embankment in front of the storm tide barrage construction, which is located at about 150 m to the right (M Thw: Mean high tide water level). (b). Landward aerial view of the storm tide barrage at the Eider river. Source: Ulf Jungjohann, Heide, Germany.

When the Eider river storm tide barrage was constructed, bed protections were arranged both on the sea and the land side. As expected, scours developed next to the rigid bed stabilization both on the inner and outer side (figures 22(a) and (b)) [38]. Due to the given scour geometry with steep embankments the common construction methods could not be used. Therefore, geotextile containers with gravel filter material were chosen. From April to August 1993, a total quantity of approximately 48 000 geotextile containers was installed. Approximately 700 geotextile containers per day were filled on site. This required the production and preparation of nearly 4500 containers per week. The loading of the geotextile containers by means of a hydraulic dredge equipment took about 1.5 h per ship (204 containers). Three to four layers of geotextile containers were loaded into each cargo hold. The realized solution with geotextile containers is considered as an exemplary and very successful case. According to the supervisor less than 10 of the total 48 000 geotextile containers were damaged during the dumping. The negative experience gained with the previously chosen woven geotextile containers did not repeat with the nonwoven geotextile containers. An important lesson was learned in this project: Tensile strength should not be taken as the decisive main parameter for choosing a suitable geotextiles. Other criteria like filter efficiency, elongation capacity, flexibility and friction properties have likewise to be taken into account.

### 4.2. Case study: ‘mega’ sand container at Narrowneck in Australia [39, 40]

**Figure 23.**
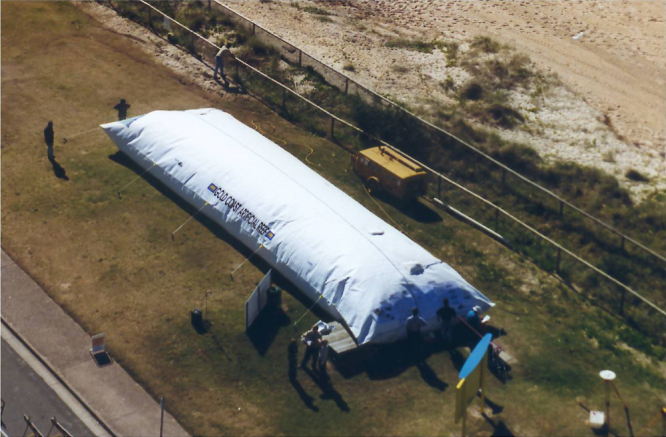
Mega sand container with a by then unprecedented size of 20 m (length) × 4.80 m (diameter) as presented in 1999 on an open day for the public at the construction site [39, 40].

The Gold Coast beaches (on the east coast of Australia) are one of the most popular surfing areas in the world. They play an important role to tourism and the economy. As the coastlines are subject to heavy erosion and therefore to a continuous loss of sand, the long-term coastal protection program ‘Gold Coast Beach Protection Strategy’ comprised—besides beach stabilizing measures—the construction of a submerged breakwater reef constructed of more than 400 ‘mega’ sand containers, which were located directly in the near shore zone and which had to be installed individually and accurately. The cross section profile of the 350 × 600 m large, V-shaped artificial reef is between 1 and 10 m below the mean sea level. It is located 200 m offshore and produces left and right hand refraction of waves. The mega sand containers were manufactured with lengths of about 20 m and diameters, which were between 3.0 to 4.8 m (up to 250 m^3^ volume and a filled weight of up to 500 t, figure 23). The containers were placed in three layers of varying size. The decisive criteria for choosing a geotextile container technology were not only cost benefits and a higher degree of functionality by using needle-punched nonwovens geotextiles but also the reduction of the risk of injuries to overturned and submerged surfers. The pre-fabricated container was spread out in the load area of the hopper's hold and was then filled via the trailing suction arm of the dredging device. Subsequently, the hopper was maneuvered to the reef area navigated via GPS to the planned installation position. The mega sand container was dropped to the proposed position on the sea bed by opening the split hull hopper in the ship's longitudinal direction. Up to 10 mega sand containers could be installed per day. This construction has already resisted some heavy seas. It took only a few months to develop a considerable ‘reef habitat’ (flora and fauna).

### 4.3. Case study: cliff erosion protection with wrapped geotextile sand cushions [41].

**Figure 24.**
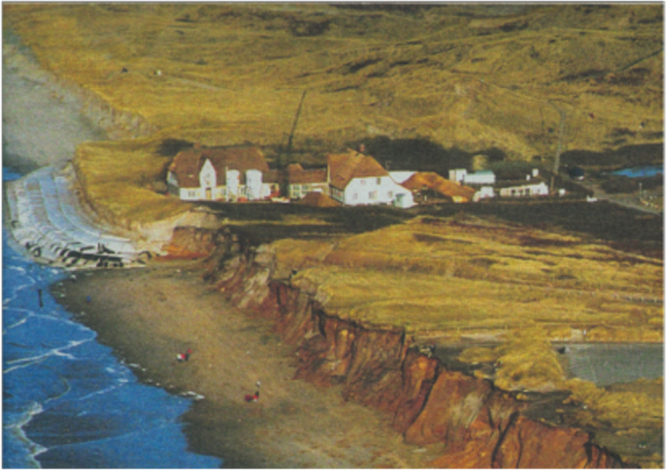
Island Sylt, 1999. Geotextile sand cushions successfully defended the historic house ‘Kliffende’ against storms, which strongly eroded the cliffs on the north and south sides of the sand cushion barrier [61]. Source: V Frenzel, Sylt-Picture.

After a series of storm tides in 1990 which had caused severe erosion problems at the western coast of the island Sylt in Germany, the historic house ‘Kliffende’ was at risk to being lost to the sea during further tidal storm surges. The authorities rejected hard rock structures such as concrete revetments at the natural sandy beaches. On this basis, the consulting engineers developed a new system consisting of geotextile sand cushions, i.e. sand wrapped into geotextiles. The geotextiles function as a filter-effective protection against sediment wash-out and the terraced layered geotextiles act in addition as reinforcement for stabilization of the dune embankment. A needle-punched composite consisting of a polypropylene slit film woven and a polyester nonwoven was used. In combination with ‘beach nourishment’ with sand this soft coastal construction was designed as ‘second defense line’. Therefore, the structure has to be re-covered by sand after severe eroding storm periods (figure 24). The total height of the construction is 8 m (inclination of 2H:1V and 4H:1V). The seaward faces of the sand cushions were accurately formed with the aid of temporary concrete shuttering elements. Parts of beach nourishment were mechanically re-deposited on the geotextile sheets and compacted. The geotextile sheets were folded up- and backwards. The sand cushions were installed on top of each other to form a stabilized beach section. Sand trap fences made of bushes were installed and beach grass served as stabilization. Thus, the final construction looked like a natural dune and survived the winter storms despite repeated exposure and direct wave attack. The sand cushions even survived a large storm in December 1999 with a water level at 2.5 m above average and wave height exceeding 5.0, showing superior effectiveness compared to all other structures being used on the island (figure 24).

### 4.4. Case study: scour protection at the Offshore Wind Farm Amrumbank West, Germany [42]

**Figure 25.**
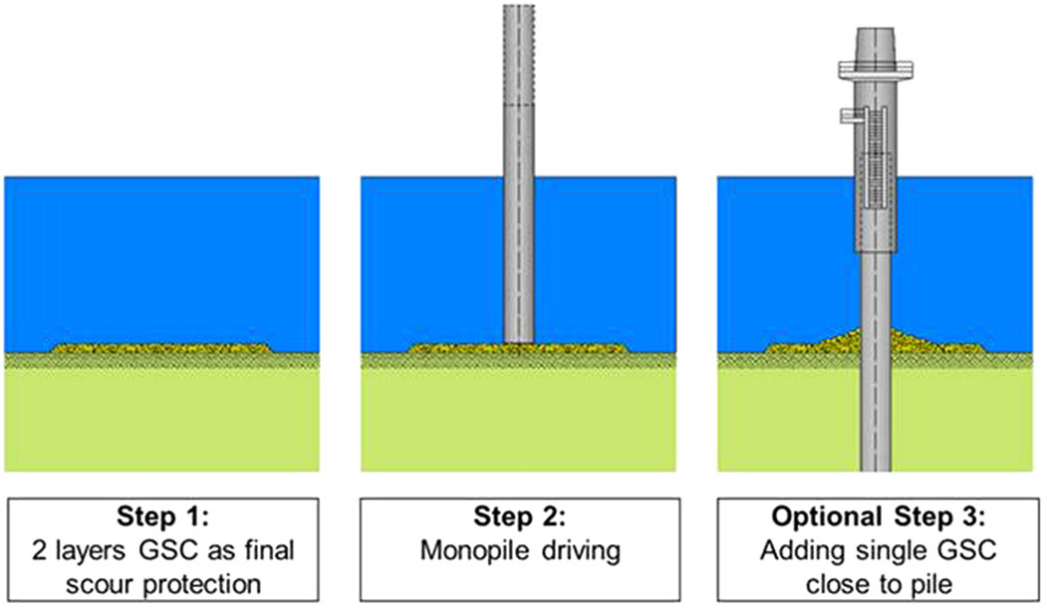
Schematic illustration: scour protection with nonwoven geotextile sand container and installation of monopile foundation of off-shore wind energy turbines [42].

The erosion of the seabed surface by currents around the underwater foundation of marine structures is a basic and severe construction problem. This applies especially to offshore wind farms composed of many wind energy turbines. The Offshore Wind Farm Amrumbank West with a total of 80 wind turbines is located 35 km northwest of Helgoland with water depths of 19 m up to 24 m. The subsoil close to the surface mainly consists of sand with varying amounts of fine, medium and coarse sand. The original erosion protection design for the turbine foundations was realized as a rock scour protection made of large stones, which piled up to a total height (filter layer and cover layer) of 2.4 m. With such a stone layer, it was not possible to realize a ‘drive through’ construction technique for the foundation pile (monopile) of the turbine. As an alternative, geotextile sand containers (made from needle-punched nonwoven geotextiles) in two layers were installed. The unfilled sand containers had a dimension of 1.45 m × 2.38 m (width and length). A volume of 1 m^3^ of sand could be filled into each. Because of the performance and handling, the filling degree was 80 vol%, which gave a weight of 1400 kg per sand-filled container. The geotextile sand containers were placed on the seabed to prevent the sea current from washing away the sand around the pile foundations of the wind turbines, thus ensuring turbine stability. They were filled on the island of R⊘m⊘ (Denmark) already 8 months prior to the construction of the basic foundation pile. To achieve a complete covering of the sea bed, the installation of two layers with geotextile sand containers was required. The installation of the wind turbine foundation started in December 2013 by driving the monopile with a diameter of 6 m through the erosion protection made of the geotextile sand containers (figure 25). Contrary to rock scour protection the geotextile scour protection could already be completely installed prior to the pile driving. Therefore, the use of geotextile sand containers logistically decoupled the installation of the scour protection from the installation of the foundation piles. The turbines are currently installed. So far, the scour protection has functioned well under severe storm impacts (extreme hurricane ‘Christian’ (October 2013) and ‘Xaver’ (December 2013)).

## 5. Economic benefits and ecological side effects

### 5.1. The geosynthetic market

Koerner from the Geosynthetic Institute in Philadelphia estimated the figures given in table 2 of the worldwide sales of geosynthetics for the year 2010 [43]. The Freedonia Group Inc., a Cleveland-based industry market research firm, published a market report analyzing the global demand and the growth in demand for geosynthetics (table 3) [44]. After a period of continuous growth in the last decades of the 20th century, starting at about 1970, and in the first decade of the 21st century, the turmoil of the worldwide financial crisis 2007–2009 caused serious problems to the geosynthetics industry. The Geosynthetics Magazine reported that prior to 2009 the US/Canadian geosynthetics market grew 5 to 6% per year before the economic debacle of 2009. In 2009, the use of geosynthetics declined about 5% in the US and Canada; but growth rebounded to about 2% in 2010. The significant decline in Western Europe in the period of 2007–2012 is likewise due to the consequences of the crisis. The development is significantly different in different European countries. Only a few detailed figures are available. In Germany, for example, well above one million of square meter of geocomposite drains alone will be installed in one special application, namely the construction of landfill capping systems in the years 2014 and 2015. However, many countries in Europe have still to deal with the aftermath of the economic crisis in the coming years.

**Table 2.** Estimate of worldwide sales of geosynthetics [43, 55].

Type	Amount (millions of m^2^)	Price (US dollars m^−2^)	Sales (millions of US dollars)
Geotextiles	1400	0.75	1050
Geogrids	250	2.50	625
Geonets	75	2.00	150
Geomembranes	300	6.00	1800
Geosynthetic clay liner	100	6.50	650
Geofoam	5	75.00	375
Geocomposite	100	4.00	400
Overall	2230		5050

**Table 3.** World geosynthetics demand (in millions of m^2^) and percentage of annual growth (market report of the Freedonia Group Inc. [44]).

Country	2007	2012	2017	2007–2012 (% annual growth)	2012–2017 (% annual growth)
Geosynthetic demand	2801	3400	5200	4.0	8.9
North America	923	965	1300	0.9	6.1
Western Europe	668	615	725	−1.6	3.3
Asia/Pacific	723	1200	2330	10.7	14.2
Central and South America	124	160	220	5.2	6.6
Eastern Europe	248	305	405	4.2	5.8
Africa/Mideast	115	155	220	6.2	7.3

Nevertheless, it is projected that annual growth will recover to about 3% in Western Europe leading to 725 million square meter of geosynthetics sales in 2017, while world-wide demand should rise to impressive 8.9% giving a global demand of more than 5 billion square meter in the year 2017. According to the report, the gain will result from ‘a much improved environment for the construction of structures and roads. Additional growth will be driven by increased market penetration, stimulated by growing concerns regarding environmental protection and greater awareness of the performance advantages of these products in a variety of applications’ and it is driven by the development in the Asia/Pacific region, especially by the developments in the Chinese and Indian markets. This region should become in 2017 the largest geosynthetics market with a share of the global market of 35% outperforming the North America regional market with a projected share of 28% in 2017. However, the United States market will probably remain the world’s largest national market contributing with 23% to the total sales of geosynthetics.

Recently, the issue of cost savings, which can be directly attributed to the use of geosynthetics in the construction of civil works features such as roads, embankments, retaining structures, erosion control features, drainage systems, reservoirs and waste containment systems, was reviewed by Christopher [45]. He identified four cost saving benefits: (1) immediate savings through substitution or reduction of selected soil materials. (2) Immediate savings through ease of installation and/or increased speed of construction. (3) Life cycle cost savings through improved performance as measured by increased longevity or reduction of maintenance. (4) Improved sustainability in terms of conserving natural environments as compared to alternate designs. Substantial cost saving may be achieved related to all four benefits. For example, the cost benefits in the above mentioned alternative package of the construction of the Saemangeum Sea Seawall in Korea, where geocontainers were used as an alternative design to the conventional rock-fill berms of the dike, was calculated to be about 6.2 million US dollars including all costs of materials and installation [35].

In Germany, according to the German landfill ordinance, geosynthetics, polymers and leak detection systems, which are used for landfill liner and capping systems have to be certified for this application (figure 2). BAM Federal Institute for Materials Research and Testing is the mandated certification authority [46, 47]. For a certification, it has to be demonstrated that the geosynthetic product will fulfill its function over a period of at least 100 years. Leak detection systems for convection barriers have to retain their function for a period of at least 30 years. To accomplish this requirement all possible external impacts and interactions between components of the system have to be taken into account.

### 5.2. Positive and negative environmental side effects

Beside the economical benefits, there are considerable positive environmental ‘side effects’ as was shown by various studies. Wallmann (back then ETH Zürich, Switzerland, now Chalmers University of Technology in Sweden) and coworkers (back then ESU-services Ltd, now most of them with treeze Ltd, Switzerland) quantified on behave of the European Association of Geosynthetic Manufacturer the environmental performance of geosynthetics compared to commonly applied materials (concrete, cement, lime or gravel) [48]. The comparison was based on life cycle assessments according to ISO 14040 and 14044 using the following impact indicators: cumulative energy demand, global warming potential of greenhouse gas emissions measured in carbon dioxide equivalents, ozone formation, particulate formation, acidification, eutrophication, land competition and water use. Four cases were analyzed and evaluated. Among these cases was the comparison of a geocomposite drain versus a conventional gravel drainage layer. The mass per area of the polymeric drain core was assumed to be 500 g m^−^^2^, which is possibly a bit ‘underdesigned’. The gravel drainage was specified according to the requirements of the European Landfill Direction and a layer of 16–32 mm gravel with thickness of 50 cm was assumed. However, such a system, even though required by the European Landfill Direction, is certainly ‘overdesigned’ [49]. Nevertheless, the comparison of geocomposite drain versus gravel drainage showed the significant advantage of the geosynthetic construction, which led to a lower impact in all indicators, except land competition, which was about the same in both cases. The non-renewable cumulative energy demand of the construction and the disposal of one square meter of drainage layer was 194 MJ (gravel layer) and 86 MJ (geocomposite drain). The cumulative greenhouse gas emissions amounted to 10.9 kg CO_2_-eq. (gravel layer) and 3.6 kg CO_2_-eq. (geocomposite drain). Lower environmental impacts of constructions using geosynthetics were likewise found in the other evaluated cases.

One of the most significant advantages is related to transportation and handling. G. Heerten reported about a landfill project of 32 ha size, where a geosynthetic capping system (geosynthetic clay liner, geomembrane, geocomposite drain) was realized instead of a conventional system (compacted clay liner, geomembrane, gravel drainage) [50]. Instead of 21 000 truckloads of clay and gravel only 165 truckloads of geosynthetic rolls were necessary. The overall cost reduction was 30% and one year of construction time was saved. Transport and handling strongly influence the carbon footprint of geosynthetic versus conventional construction products. The carbon footprint is the amount of emitted greenhouse gas (in carbon dioxide equivalences) related to the life cycle of the product. Table 4 gives some additional results.

**Table 4.** Summary of case studies on cumulative energy demand^a^ (CED) and carbon dioxide foot print [45].

Project	Geosynthetic and alternative approach	CED GJ	CO_2_ tonnes	Reference
New roadway embankment near Frankfurt, Germany	Geosynthetic reinforced soil structure	1350	101	
	Reinforced concrete wall	4549	542	[45]
District road K34 near Aachen, Germany	Geogrid subgrade stabilization	1182	49	
	Lime subgrade stabilization	6383	1325	[45]
External Sealing of Kinzig River Dike, Germany	Geosynthetic clay liner	2585	145	
	Compacted clay liner	4403	357	[57]

a For terms and definitions, see the guideline VDI4600 of the Association of German Engineers (VDI).

Having discussed the environmental benefits, we should not neglect to consider possible negative side effects. These are related to two issues: plastics in the environment, especially in the ocean, and emission of additives and their degradation products from the polymeric material into the environment [51–54], which might be unhealthy or may have some ecotoxic effects. The issue of plastic debris in the ocean has found considerable public attention and has been the focus of campaigns of many environmental organizations. Impressive movies and pictures were distributed over the media. However, it is difficult to obtain reliable data. Recently, a scientific effort was undertaken by a large group of experts to estimate the magnitude of floating plastic debris in the open ocean and to discuss the fate of the polymeric particles [55]. A conservative high estimate gave a total load in the surface waters of the ocean of 35.2 kilotonnes of plastic debris. This figure may be compared with the amount of plastics, which was used, for example, in the gigantic project of the Saemangeum seawall mentioned above. With 10 m circumference, 20 000 m overall length, 0.005 m thickness and about 1 kilotonnes m^−3^ density as rough estimates of the properties of the geotextile tubes, we obtain a total amount of 1 kilotonne of installed polymeric material, which is a small but significant fraction of the overall plastic debris in the ocean. At some time in the future, the geosynthetic material will become brittle due to aging. The product will rupture and disaggregate. However, this is primarily not an environmental but a technical problem, because failure of the whole construction will be triggered by such degradation processes. Maintenance has to identify aging effects at an early stage and the retrieval of all synthetic components has to be an important part of the reconstruction, which will become necessary in a remote future. Therefore, the issue of the occurrence of plastic debris due to the use of a large amount of geosynthetics in hydraulic and coastal engineering is closely related to the question, whether the lifetime of the geosynthetic products actually fits to the envisaged service life of the whole construction. The answer to this question strongly depends on the properties of the used polymer and its additive package. For example, a polypropylene nonwoven geotextile properly stabilized with hindered amine antioxidants of high molecular weight, which is applied in an underwater construction, where the supply of oxygen is limited and the temperature is constantly on a low level, will last at least for one hundred years. However, the same product will fail within a few decades if poor antioxidant packages are used.

The same reasoning applies to the environmental impacts of the emission of additives. The loss of additives, e.g. of antioxidants from polyethylene or polypropylene and of plasticizer from polyvinylchloride, is intimately related to the aging of the geosynthetic product. In this respect, the depletion processes as studied by material scientists and their results should be evaluated with respect to health concerns. The concentration of antioxidant is very low (∼10^−1^ wt%). Many of them are used in food packaging [56]. Safety values and respective health considerations are available with respect to this field of application. The concentrations of plasticizer may be quite large (∼10^+1^ wt%) and some of them may cause damage to human health. Many substances are registered and evaluated according to the REACH (registration, evaluation, authorization and restriction of chemicals) regulations at the European Chemicals Agency. Useful information might be obtained from this data source. For example, most of the produced plasticizers, which are used to make polyvinylchloride soft and flexible, are out of the class of phthalate substances. It is planned by the EU to phase out the so-called ‘low phthalates’, which were classified by REACH as CMR substances (carcinogenic, mutagenic or toxic to reproduction), with the beginning of 2015. The far less risky so-called ‘high phthalates’, which are commonly used nowadays, are registered for REACH and are not considered as substances of very high concerns.

## 6. Summary and conclusions

Geosynthetics are planar polymeric products, which are used in connection with soil, rock or other soil-like materials to fulfill various functions in geotechnical and geoenvironmental engineering. Development of these products started after the Second World War. Since then, they became one of the outstanding innovations in geotechnical engineering with ever-growing importance in the construction industry. Geosynthetics are especially used in geoenvironmental engineering. Our review focused on two fields of application.

(1) Examples of material problems were discussed with respect to the use of geocomposite drains and geogrids in landfill capping systems. Like all polymeric products, geocomposite drains are susceptible to creep and rupture due to creep. The geocomposite drain is slowly and continuously deformed under long lasting shear and pressure forces. The thickness is reduced and thereby the drainage capacity. If the in-plane deformation reaches a critical value in the course of time, shear failure might occur. Likewise, if the thickness reaches a critical value the structure of the drain core might collapse. These effects were shown using data provided by the manufacturers of geocomposite drains. It was illustrated how the long-term water flow capacity and the acceptable limits of shear stress and normal stress (pressure) with respect to shear rupture and drain core stability as well as the lifetime with respect to these failure modes can be determined using standard test methods. The design of long lasting geotechnical structures has to take into account these specific characteristics of the long-term performance of geocomposite drains.

Geogrids, which are installed as part of landfill barrier systems to prevent sliding failure on long and steep slopes, have to be safely anchored. The design and calculation of the anchorage is based on simple design rules. Basically, it is assumed that the pull-out resistance is proportional to the shear strength of the backfill in the anchoring, the vertical load and the anchoring length. The physical assumptions and limitations of this basic rule were discussed. For those geogrids, for which the mobilization of a passive thrust of the soil in and near the geogrid openings by the displacement of the transverse grid elements substantially contributes to the pull-out resistance, the mechanical strength of the junction between LEs and TEs is of crucial importance. The relation between mechanical properties of the junction, the flexibility of the longitudinal grid elements, the surface friction and the finally achieved pull-out resistance was exemplarily shown by a model calculation. Due to aging and creep the long-term junction strength may be significantly lower than the short-term strength. We concluded that in the long run, there is a certain critical pull-out resistance and an associated critical anchorage length. Both are determined by the strength of the junction embedded into the soil. For a safe design, it is not allowed to go beyond that limit. This requirement restricts the application range of the common design rule.

(2) The second field of application, which we considered, is the field of coastal and hydraulic engineering. We illustrated by various case studies how the use of geosynthetic containers filled with sand or other soil materials can replace more expensive constructions made of rock, steel or concrete to protect against erosion and scour formation. Coastal protection and hydraulic engineering with geotextile containers is a very active field of applications. Projects of small and very large scale have been realized and are still under evaluation. However, hitherto they functioned well even under severe impacts of heavy storms.

Related to the use of geosynthetics in geoenvironmental engineering are positive and negative environmental side effects. The positive effects are essentially due to the ‘carbon footprint’ of these products. The products are light and can be easily handled and transported. Therefore, the use of geosynthetics may substantially reduce the emission of the greenhouse gas (as measured in carbon dioxide equivalences) throughout the whole construction process. Environmental concerns refer to the accumulation of plastic debris in the environment after the end of life of the constructions and to the emission of ecotoxic additives in the plastic material into the environment. Both problems can be solved by an appropriate choice of the basic polymeric materials used for the geosynthetic products.

The technical, environmental and cost benefits of geosynthetics can only be realized and the drawbacks avoided, if (1) the polymers and their additives are adequately chosen, above all with respect to their long-term behavior, (2) the products are well designed with respect to the envisaged function and (3) the construction design is realized by an engineer well informed and educated in the specific properties of the products. Violating these principles might easily lead to failure. However, such reasoning is common in the application of advanced materials.
